# Thoracic Ultrasound–Related Management Change: Predictors and the Role of Operator Certification (Secondary Analysis of UltraMAN)

**DOI:** 10.1002/jcu.70104

**Published:** 2025-10-16

**Authors:** Jorge E. Lopez Matta, Micah L. A. Heldeweg, Luigi Pisani, Carlos V. Elzo Kraemer, Stefanie Slot, Mark E. Haaksma, Jasper M. Smit, Amne Mousa, Giovanna Magnesa, Fabrizia Massaro, Hugo R. W. Touw, Viviane Schouten, Pieter R. Tuinman, David J. van Westerloo

**Affiliations:** ^1^ Amsterdam Leiden Intensive Care Focused Echography (ALIFE) Amsterdam the Netherlands; ^2^ Department of Intensive Care Medicine Leiden University Medical Center Leiden the Netherlands; ^3^ Department of Intensive Care Medicine Amsterdam University Medical Centers Amsterdam the Netherlands; ^4^ Department of Intensive Care Medicine Regional General Hospital F. Miulli Acquaviva delle Fonti metropolitan city of Bari Italy; ^5^ Department of Intensive Care Medicine Noordwest Ziekenhuisgroep Alkmaar the Netherlands; ^6^ Department of Intensive Care Medicine Radboud University Medical Hospital Nijmegen the Netherlands; ^7^ Amsterdam Cardiovascular Sciences Amsterdam UMC, Vrije Universiteit Amsterdam Amsterdam the Netherlands; ^8^ Amsterdam Institute for Immunity and Infectious Diseases Amsterdam UMC, Vrije Universiteit Amsterdam Amsterdam the Netherlands

**Keywords:** cardiovascular disease, clinical decision making, critical care, POCUS, thoracic ultrasound

## Abstract

**Objectives:**

To investigate which patient characteristics, ultrasound operator certification level, and thoracic ultrasound (TUS) examination findings are associated with a TUS‐induced change in clinical management in adult intensive care unit (ICU) patients.

**Design:**

Post hoc analysis of a prospective international observational study (UltraMan study) on the impact of thoracic ultrasound on clinical management of critically ill patients. The first TUS examinations of each patient included in the study were included in this analysis. Multivariable logistic regression was performed to identify which patient characteristic(s), operator certification level, or TUS‐related factors were significantly associated with a change in management.

**Interventions:**

None.

**Measurements and Main Results:**

The first TUS examinations of each of the 534 patients were included in this analysis. TUS led to management changes in almost half of the patients in whom a TUS was performed (44.6%). TUS‐induced management changes were significantly associated with patient characteristics. Specifically, a medical history of cardiovascular disease demonstrated a significant association (OR: 1.73; 95% CI: 1.12–2.68). In terms of TUS examination findings, hypovolemia demonstrated a significant association with a change in management (OR: 2.05; 95% CI: 1.10–3.80). No significant association was found between ultrasound operator certification level and changes in management driven by TUS.

**Conclusions:**

This study indicates that TUS was associated with management changes in 44.6% of ICU patients, with stronger associations in those with cardiovascular disease and hypovolemia, and no detectable effect of operator certification in adjusted analyses. As a post hoc analysis of an observational cohort, these findings warrant cautious interpretation and underscore the importance of competency‐based training and quality assurance.

## Graphical abstract

Thoracic ultrasound frequently altered ICU management decisions. Changes were more likely in patients with cardiovascular disease or hypovolemia and occurred independently of operator certification. These findings support the routine use of TUS in critically ill patients, even when performed by less‐experienced but supervised operators.

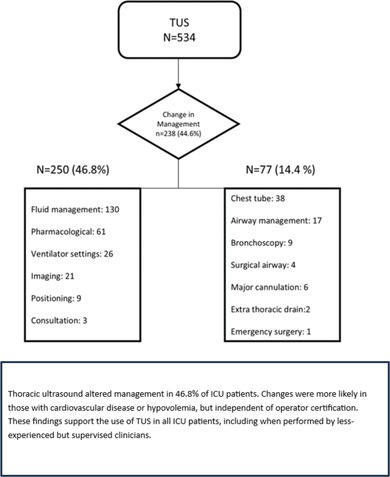

## 1 Introduction

Point of care ultrasound (POCUS) allows for rapid diagnosis and management of critical illnesses, making it an essential tool in the intensive care unit (ICU) (Lichtenstein and Meziere 2008; Malbrain et al. 2020). It can be used to evaluate different organ systems, including the lungs, heart, abdomen, and vascular system, and provides valuable information that may guide and impact clinical decision‐making in the ICU (Volpicelli et al. 2012; Heldeweg et al. 2021).

However, it is unclear if POCUS and specifically thoracic ultrasound (TUS) should be a standard diagnostic tool that can be used in all patients, or if there are subgroups of patients that may benefit more from TUS since they may have an increased likelihood of TUS‐induced management changes as compared to other patients (Zieleskiewicz et al. 2015; Qaseem et al. 2021). Insight into factors that may increase the likelihood of management changes based on TUS may help clinicians make more informed decisions in the care of their patients at the bedside, thereby allowing a more efficient workflow and performing TUS only in those patients that have a high likelihood of TUS‐related management changes. On the other hand, it may be assumed that TUS may be less impactful in patients with less optimal ultrasound echogenicity, such as obese patients, or in settings where the clinical staff is less experienced in TUS.

The main aim of this study is to investigate which factors are related to an increased or decreased likelihood of TUS‐induced management changes. We hypothesized that TUS‐induced management changes may be positively and negatively associated with certain patient characteristics, as well as positively related to ultrasound operator experience as well as specific actionable TUS findings (e.g., lung edema, signs of pneumothorax, and pleural effusion).

## 2 Material and Methods

### 2.1 Study Design

This is a post hoc analysis of the UltraMan study, an international prospective observational study conducted in the ICU of two academic and two large non‐university hospitals in the Netherlands and Italy (Heldeweg et al. 2023). Ultrasound examinations were conducted by physicians whenever there was a clinical indication for TUS. The study was approved by the institutional review board, who had previously waived the need for written informed consent.

The examination focused on assessing respiratory, circulatory, or volume status, or a combination of these factors. Rather than describing the examination itself, the operator completed a standardized case report form (CRF) before and after the examination, as outlined in Supplemental Digital Content 1. The CRF included details on the operator and their training level, the reason for the TUS examination as well as the patient's current diagnosis and treatment plan. After the examination, post‐ultrasound questions in the CRF were completed, including the TUS findings, the clinical contribution of TUS, and whether the performance of a TUS had resulted in changes in diagnosis and/or management. The TUS semiotics and finding definitions were consistent with current international recommendations (Qaseem et al. 2021). All CRF data were transcribed into an electronic data capture system.

### 2.2 Population

Adult ICU patients (> 18 years) who underwent a clinically indicated TUS examination. In the current analysis, only the first ultrasound examination per patient was analyzed to more precisely analyze whether any correlation exists between patient‐related characteristics, operator factors, and TUS findings with a change in management.

### 2.3 Variables and Outcomes

Patient‐related characteristics included: gender and body mass index (BMI). The reason for hospitalization was divided into elective surgery, emergency surgery, trauma, or medical. Previous medical history was extensively noted. This analysis focused on cardiovascular and pulmonary medical history, as medical diagnoses in these organ systems were deemed most likely to significantly influence thoracic ultrasound (TUS) findings. Patients with a cardiovascular medical history had either one or more of the following conditions: peripheral artery disease, atrial fibrillation, ischemic heart disease, valvular heart disease, diabetes mellitus, hypertension, hypercholesterolemia, thoracic aortic aneurysm, abdominal aortic aneurysm, left‐sided heart failure, right‐sided heart failure, and deep venous thrombosis. Pulmonary medical history was noted if the patient had one or more of these conditions: chronic bronchopulmonary disease (COPD), asthma, pneumonia, pulmonary embolism, lung cancer, and COVID‐19 infection.

Since we postulated that operator experience might impact TUS‐induced management changes, we recorded whether the operator had completed a basic teaching program for TUS and was certified for this ultrasound skill (yes/no). Certification definitions varied across participating centers. In the largest contributing centers (VUmc and LUMC), examinations performed by non‐certified clinicians were routinely discussed with, and when needed supervised at the bedside by, a certified intensivist before the findings were used to guide patient management. Where available, images/clips were archived to the picture archiving and communication system (PACS). Where the ICARUS program was used, an operator was considered certified after completing the two‐day ICARUS course (basic + consolidation), documenting ≥ 40 complete thoracic ultrasound examinations in a supervised logbook, and passing a formal practical assessment, resulting in certificate issuance (see Supplementary Methods S2). Programs were deemed equivalent to ICARUS if they included ≥ 2 course days with hands‐on training, supervised complete examinations with sign‐off and a formal competency assessment, and coverage of heart–lung POCUS including IVC/volume‐status assessment, documentation, and archiving comparable to ICARUS (Touw et al. 2015). At the largest contributing centers, supervising certified intensivists typically had > 10 years' clinical ultrasound experience. Non‐certified operators typically acquired TUS skills through institutional POCUS teaching (on‐service sessions and local curricula), bedside mentorship, and supervised clinical practice; years since first TUS and cumulative operator case volume were not captured across centers.

Some ultrasound findings may prompt more immediate action and may thus be linked to a greater likelihood of instigating management alterations. Because some ultrasound findings may prompt immediate action, we recorded the following sonographic findings: inferior vena cava (IVC) collapse (Corl et al. 2017; Lau and See 2022), lung edema (Lau and See 2022), pleural effusion (Cotton et al. 2018; Hansell et al. 2021), atelectasis (Hansell et al. 2021), pneumonia (Hansell et al. 2021; Alzahrani et al. 2017), acute respiratory distress syndrome (ARDS) (Lichtenstein et al. 2004), pneumothorax (Lau and See 2022), diaphragmatic dysfunction (Kim et al. 2011) as well as gross cardiac pathology in the form of valvular or functional abnormalities as well as pericardial effusion (Lau and See 2022). We also captured composite interpretations of hypervolemia, hypovolemia, and hyperdynamic state, defined as follows: Hypervolemia: pleural effusion and/or B‐line–predominant lung profiles with non‐collapsible IVC. Hyperdynamic state: markedly hyperkinetic left ventricle (LV) on standard windows. ‘Hypovolemia’: small/collapsible IVC and/or hyperdynamic LV. Numeric thresholds were not captured in the CRF, and these composite interpretations were based on the operators' protocol‐based judgment. Because more than one management action could follow a single TUS examination, counts of specific change types represent events rather than examinations; composition percentages use all changes as the denominator, whereas cohort‐level rates use all examinations.

Definition of TUS‐induced change in management. A management action was classified as *TUS‐induced* only when the treating team judged that the thoracic ultrasound examination materially contributed to initiating, modifying, or timing the action. When a decision had already been made based on prior imaging (e.g., chest drainage planned after CT) and TUS was used solely for procedural localization/site selection, this was not counted as a TUS‐induced change. Conversely, when TUS revealed or clarified a finding that triggered or altered the plan (e.g., confirmation of a drainable effusion uncertain on chest radiography, or identification of a pneumothorax at the bedside), the ensuing action was counted as TUS‐induced.

### 2.4 Statistical Analysis

For data processing and statistical analysis, IBM SPSS Statistics (version 27) was used. Baseline and outcome variables were presented as mean (±SD), median [IQR], and/or number and percentages (%) as appropriate. Proportions or percentages for outcomes were reported with 95% confidence intervals. Multivariable logistic regression was performed to identify which patient characteristic(s), operator certification level, or TUS‐related factors were significantly associated with a change in management. The reported results include odds ratios and 95% confidence intervals. Statistical significance was considered if the *p* value was < 0.05.

## 3 Results

**FIGURE 1:**
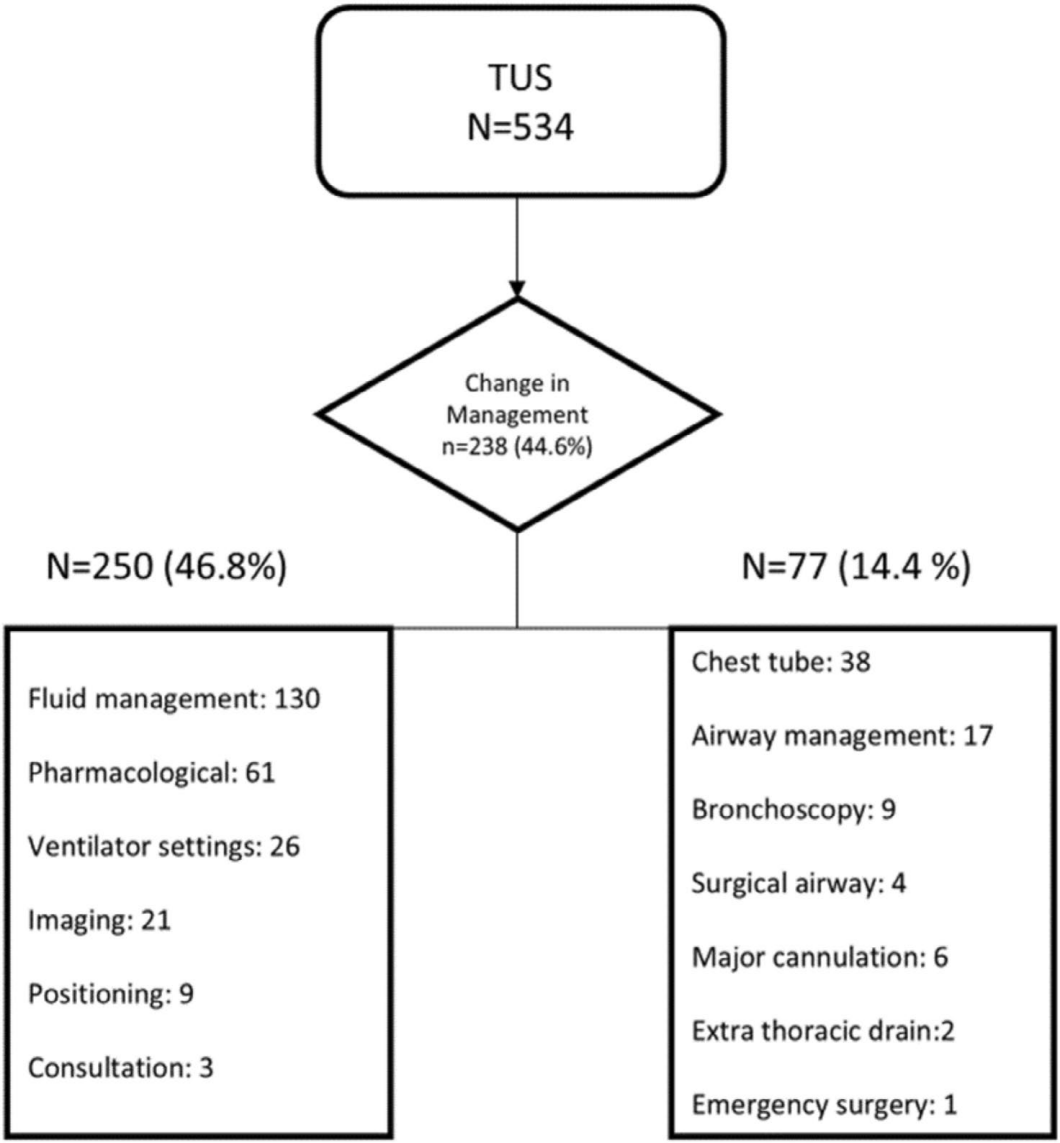
Diagnostic and management changes induced by thoracic ultrasound (TUS). Multiple changes in diagnosis or management (noninvasive and/or invasive) could occur in one patient. Airway management included intubation and extubation. Major cannulation included pulmonary artery catheter, extracorporeal membrane oxygenation, coronary angiography, and central venous cannulation. Ventilation settings included change of ventilator settings, start of high‐flow nasal cannula, and non‐invasive ventilation. Positioning included prone. The diamond shows the proportion of examinations with any change (238/534, 44.6%). Values “*N* = 250 (46.8%)” and “*N* = 77 (14.4%)” are cohort percentages per 534 examinations; items inside the boxes are *counts of change events*, and a single examination may contribute multiple events.

Of the 725 TUS examinations performed in 534 patients, the first TUS examination of each patient was included in the current analysis. Of these 534 index examinations, 279/534 (52.2%) were performed by certified and 255/534 (47.8%) by non‐certified operators; 0/534 (0%) missing. Baseline characteristics of patients are described in Table 1. In 238/534 (44.6%) of the patients in which a TUS was performed, the TUS resulted in a change in management. As multiple actions could follow one examination, we recorded 327 discrete changes in total (250 non‐invasive; 77 invasive). Non‐invasive changes accounted for 250/327 (76.5%) of all changes (i.e., 250/534 [46.8%] of examinations), with fluid management most common (130/250 [52.0%]; 130/327 [39.8%] of all changes). Invasive changes accounted for 77/327 (23.6%) (i.e., 77/534 [14.4%] of examinations), most commonly chest tube insertion (38/77 [49.4%]; 38/327 [11.6%] of all changes). The specific management changes induced by TUS are depicted in Figure 1. Multivariable logistic regression analysis failed to reveal an association between TUS‐induced change in management and specific patient factors, including gender, BMI, and reason for admission (Table 2).

**TABLE 1:** Characteristics of included patients at time of first thoracic ultrasound examination.

Baseline variables	Patients (534)
Age, years	62.5 ± 15
Gender (male)	372 (69.7%)
Body mass index	25.6 ± 7.8
Invasively ventilated	357 (66.9%)
PaO_2_/FiO_2_, mmHg	199.5 ± 107.8
Sequential organ failure assessment	8.7 (3.6)
Operator certification	
Certified, *n* (%)	279 (52.2%)
Non‐certified, *n* (%)	255 (47.8%)
Missing, *n* (%)	0 (0.0%)
By center	
VUMC—certified/non‐certified (certified % of total *n*)	107/198 (35% of 305)
LUMC—certified/non‐certified (certified % of total *n*)	117/21 (84.8% of 138)
Pisani—certified/non‐certified (certified % of total *n*)	11/35 (23.9% of 46)
NWZ—certified/non‐certified (certified % of total *n*)	44/1 (97.8% of 45)
Medical history	
None	54 (10.1%)
Cardiovascular	326 (61.0%)
Pulmonary	133 (24.9%)
Reason for admission	
Elective surgery	58 (10.9%)
Emergency surgery	40 (7.5%)
Trauma surgery	64 (12.0%)
Medical	356 (66.7%)
Other	16 (3.0%)
COVID‐19	103 (19.3%)
Length of stay (d)	7.8 (13.4)

*Note:* Variables were presented as means ± SD, medians[IQR], or *n* (%) depending on distribution.

**TABLE 2:** Multivariable logistic regression of TUS‐induced change in management and patient characteristics, operator certification, and TUS findings.

	Multivariable logistic regression		
	*p*	OR	95% CI
Patient characteristics			
Gender	0.55	1.13	0.75–1.71
BMI	0.921	0.99	0.96–1.03
Reason for admission			
Elective surgery^a^		1.00	
Emergency surgery	0.84	0.91	0.36–2.30
Trauma surgery	0.27	1.59	0.70–3.65
Medical	0.10	1.66	0.91–3.03
Other	0.99	0.99	0.29–3.36
Medical history			
Cardiovascular general	0.01^b^	1.73	1.12–2.68
Pulmonary general	0.65	0.90	0.58–1.40
None	0.59	0.82	0.40–1.69
Operator			
Certification (yes)	0.90	0.98	0.67–1.42
Ultrasound findings			
No findings	0.34	0.71	0.35–1.44
Collapsed vena cava	0.50	1.48	0.48–4.55
Hypervolemia	0.67	1.28	0.41–4.04
Hypovolemia	0.02^b^	2.05	1.10–3.80
Hyperdynamic	0.21	2.94	0.54–16.00
Cardiac dysfunction	0.08	1.76	0.94–3.29
Pericardial effusion	0.82	1.15	0.34–3.86
Cardiac valvular pathology	0.44	0.40	0.04–4.11
Lung edema	0.70	1.11	0.66–1.87
Pleural effusion	0.40	1.22	0.77–1.95
Atelectasis	0.71	1.09	0.69–1.73
Pneumonia	0.93	0.98	0.57–1.66
ARDS	0.86	0.61	0.30–1.28
Pneumothorax	0.09	2.34	0.88–6.22
Diaphragmatic dysfunction	0.28	0.41	0.08–2.10

^a^ Elective surgery’ serves as the reference category for the “reason for admission” variable. Odds ratios for other admission reasons are computed relative to “elective surgery.”

^b^ *p*‐value < 0.05.

However, as observed in Table 2 significant associations between TUS‐induced changes in management and the medical history of patients were found. Specifically, the presence of a medical history of cardiovascular disease was related to a higher chance of finding TUS‐induced changes in management (OR 1.73; 95% CI: 1.12–2.68).

TUS‐induced changes in management and operator certification showed no significant association (OR: 0.98; 95% CI: 0.67–1.42) (Table 2). Regarding specific ultrasound findings, the analysis only revealed a significant positive association with hypovolemia (OR: 2.05; 95% CI: 1.10–3.80). The adjusted odds ratios from this analysis are also shown in Figure S1.

Exploratory outcomes (unadjusted) are shown in Table 3. In‐hospital mortality was 85/238 (35.7%) in the TUS‐induced changes in management group versus 80/296 (27.0%) in the No‐TUS‐induced changes in management group; invasive mechanical ventilation at the time of index TUS occurred in 162/238 (68.1%) versus 197/296 (66.6%); length of stay (days), median [IQR], 2.0 [1.0–11.0] versus 3.0 [1.0–10.0].

**TABLE 3:** Clinical outcomes by TUS‐induced management change (unadjusted, complete cases).

Outcome	Change group (*n*/*N*, %) (*N* = 238)	No‐change group (*n*/*N*, %) (*N* = 296)
In‐hospital mortality	85/238 (35.7%)	80/296 (27.0%)
Invasive mechanical ventilation at time of TUS	162/238 (68.1%)	197/296 (66.6%)
Length of stay, days—median [IQR]	2.0 [1.0–11.0]	3.0 [1.0–10.0]

*Note:* Change group = TUS‐induced change; no‐change group = no TUS‐induced change. LOS reported as median [IQR].

## 4 Discussion

In this post hoc analysis of our previous UltraMan study, our objective was to explore the determinants influencing whether a performed TUS directly impacted clinical management. In our ICU practice, TUS is often used alongside chest radiography and CT; our endpoint reflects the treating team's judgment that TUS materially contributed to a given action (see Methods). The major findings can be summarized as follows: the change in management secondary to TUS is independent of patient characteristics such as gender, BMI, or reason for admission. Within this category, only a medical history of cardiovascular disease was significantly associated with changes in management. Additionally, these changes were independent of operator certification. The only ultrasound finding significantly associated with changes in management was that suggesting hypovolemia.

With regard to patient characteristics, we hypothesized that factors affecting the “acoustic window” of patients, such as BMI and gender (presence of breast tissue), might be associated with a lower chance for changes in management following TUS since it is widely assumed that TUS is less reliable in these patients. In contrast to our hypothesis, we identified no differences between patient groups stratified for BMI or gender, indicating that ultrasound is useful independent of these factors. Additionally, we anticipated that greater ultrasound expertise—proxied by operator certification—would be associated with a higher likelihood of TUS‐related management changes. In adjusted analyses, we did not detect an independent association between certification and management change. Given the heterogeneity of certification across centers and the routine supervision/over‐read of examinations performed by non‐certified clinicians in the largest contributing center, this finding should be interpreted cautiously. Our data are compatible with the interpretation that well‐supervised examinations by non‐certified clinicians can yield management changes comparable to those by certified clinicians, consistent with literature linking regular ultrasound use and operator practice to clinical impact (Zieleskiewicz et al. 2015).

Conversely, a significant association, with an odds ratio of > 1, was found for patients with cardiovascular disease in the patient characteristics category and images suggesting hypovolemia in the TUS findings category. For the former, this can be explained by the fact that these patients were undergoing initial dynamic cardiac/thoracic assessments in newly developed critical conditions, a situation that may, as expected, necessitate changes in their medical management. Regarding the association with hypovolemia as a TUS finding, it is unsurprising given the ICU setting; such a finding would prompt a change in management, certainly in fluid management.

In exploratory, unadjusted comparisons of outcomes, patients with a TUS‐related change in management had higher in‐hospital mortality, similar rates of invasive mechanical ventilation at the time of the index TUS, and small differences in length of stay; this pattern is consistent with confounding by indication and should not be interpreted as evidence of benefit or harm.

This study, while informative, has several limitations outlined in the original Ultraman study. Additionally, the present study is based on a post hoc analysis not powered for certain endpoints. Certification status was captured as a binary variable across centers with heterogeneous curricula and competency assessments; numeric thresholds for some composite ultrasound interpretations (e.g., hypovolemia) were not captured in the CRF. Although sites followed their own archiving and over‐read policies, we did not quantify their frequency, limiting assessment of potential verification bias. Operator‐level experience (years and/or case volume) was not systematically recorded across centers, limiting our ability to distinguish the influence of certification from practical experience. Nevertheless, it offers valuable insights into the factors associated with clinical actions based on ultrasound in real‐world settings. The strengths of this study include its post hoc analysis of the largest study on this subject to date, enhancing external validity due to its multicenter and international design, and involvement of over 100 operators with varying levels of expertise. The use of strict registration on case report forms and electronic data capture systems reduces subjectivity and enables more robust data analysis.

## 5 Conclusion

Thoracic ultrasound (TUS) was associated with management changes in 44.6% of ICU patients, with stronger associations in those with cardiovascular disease and hypovolemia. In adjusted analyses, we found no independent association with operator certification. Given the post hoc observational design, these findings warrant cautious interpretation and underscore the importance of competency‐based training and quality assurance.

## Author Contributions

**Jorge E. Lopez Matta**, MD: conceptualized the study, drafted the manuscript, analyzed the data, and, together with all other authors, acquired the data from the original study. Contributed to the manuscript revisions and approved the final version. **Micah L. A. Heldeweg**, MD, PhD: assisted in the analysis of the data. Revised the manuscript and approved the final version. **Pieter R. Tuinman**, MD, PhD: conceptualized the study, assisted in data acquisition, revised the manuscript, and approved the final version. **David J. van Westerloo**, MD, PhD: conceptualized the study, assisted in data acquisition, revised the manuscript, and approved the final version. **Luigi Pisani**, MD, PhD, **Carlos V. Elzo Kraemer**, MD, **Stefanie Slot**, MD, PhD, **Mark E. Haaksma**, MD, PhD, **Jasper M. Smit**, MD, **Amne Mousa**, MSc, **Giovanna Magnesa**, MD, **Fabrizia Massaro**, MD, **Hugo R. W. Touw**, MD, PhD, **Viviane Schouten**, MD: contributed to data acquisition, helped revise the manuscript, and approved the final version. Guarantor: **Jorge E. Lopez Matta**, MD, takes responsibility for the integrity of the work as a whole, from inception to published article.

## Conflicts of Interest

The authors declare no conflicts of interest.

## Supporting information


**Data S1:** Supporting Information.

**Data S2:** Supporting Information.

**Figure S1:** Supporting Information.

## Data Availability

The data that support the findings of this study are available from the corresponding author upon reasonable request.
