# Supplementary figures and images for: Thoracic Ultrasound–Related Management Change: Predictors and the Role of Operator Certification (Secondary Analysis of UltraMAN)
Source: J Clin Ultrasound. 2025 Oct 16;54(3):635–41. doi: 10.1002/jcu.70104 (PMC12967746; doi:10.1002/jcu.70104)

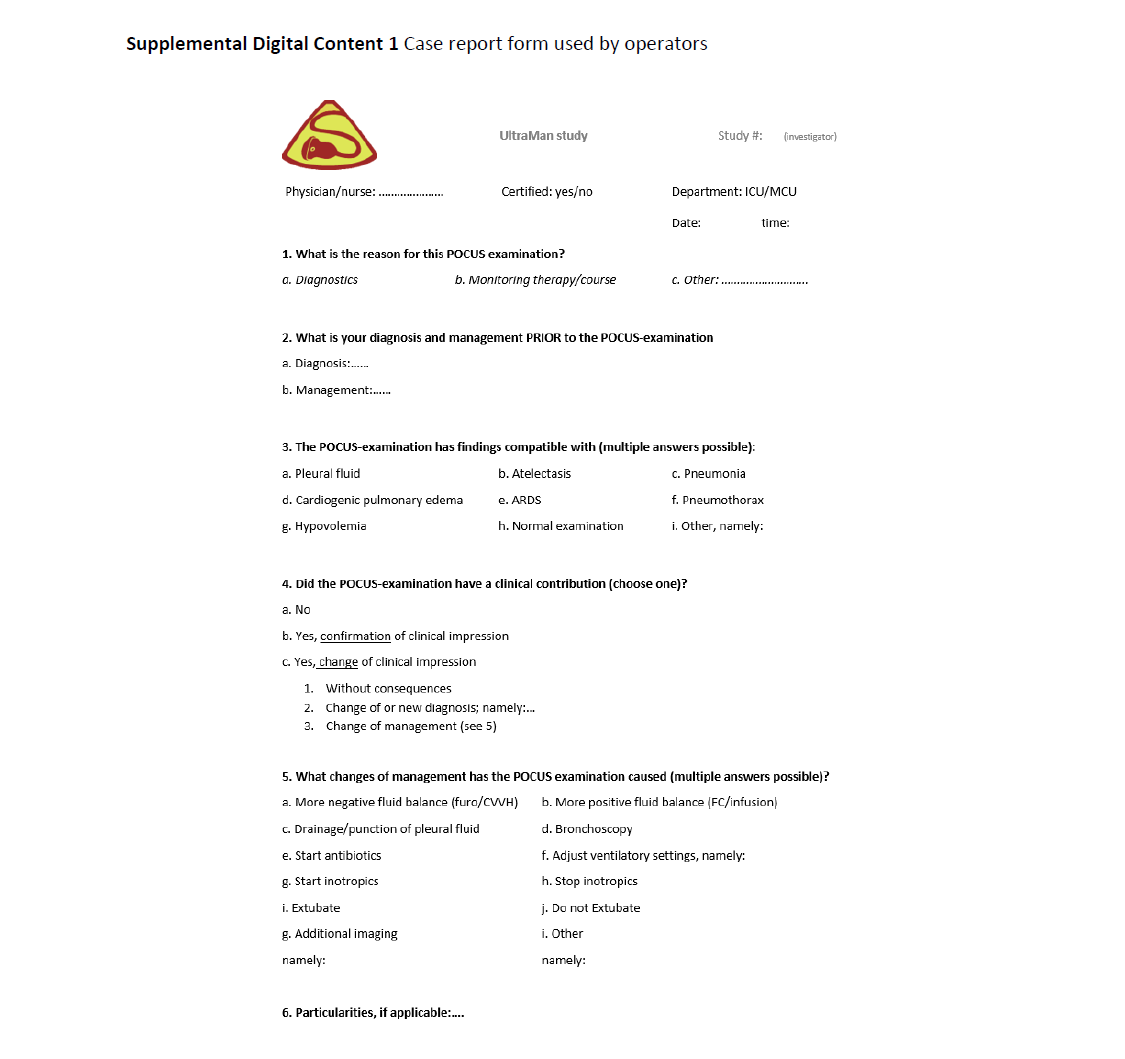

Supplement: Supplementary file 1 — Data S1: Supporting Information. [file JCU-54-635-s002.docx]
