# Supplementary material for: Thoracic Ultrasound–Related Management Change: Predictors and the Role of Operator Certification (Secondary Analysis of UltraMAN)
Source: J Clin Ultrasound. 2025 Oct 16;54(3):635–41. doi: 10.1002/jcu.70104 (PMC12967746; doi:10.1002/jcu.70104)
Supplement: Supplementary file 2 — Data S2: Supporting Information. [file JCU-54-635-s003.docx]

**ICARUS (Intensive Care Ultrasound)**

Adult Intensive Care, AmsterdamUMC location VUmc Authors B.L. ten Tusscher and H.P.M.M. Gelissen.

# Contents

Introduction.................................................................................................................................2

Definition................................................................................................................................2

ICARUS Commission ............................................................................................................2

Certification............................................................................................................................2

Status of manufactured ultrasound images...........................................................................3

ICARUS program...................................................................................................................3

Handling the ultrasound device..............................................................................................4

ICARUS Basic recordings......................................................................................................5

ICARUS – Recordings and practical instructions...................................................................5

Lung and pleura.....................................................................................................................6

Heart and blood vessels ........................................................................................................7

Indications..............................................................................................................................7

Reporting ...............................................................................................................................8

Choice of ultrasound probe....................................................................................................9

Lungs .......................................................................................................................................10

Position of the probe for lungs and pleura...........................................................................10

Interpretation of ultrasound images of lungs and pleura......................................................11

Echo profiles and the BLUE protocol...................................................................................14

Heart ........................................................................................................................................16

Position of the probe for heart and vessels..........................................................................16

Parasternal recordings.........................................................................................................16

Parasternal long axis (PLAX)..........................................................................................16

Optional...........................................................................................................................17

Parasternal short axis (PSAX).........................................................................................18

Apical images ......................................................................................................................21

AP4C...............................................................................................................................21

Optional...........................................................................................................................23

AP5C...............................................................................................................................25

AP2C...............................................................................................................................28

AP3C...............................................................................................................................28

Subcostal recordings ...........................................................................................................30

SC-4C..............................................................................................................................30

SC-SAX ...........................................................................................................................30

SC-VCI.............................................................................................................................31

Literature..................................................................................................................................33

# Introduction

Ultrasound has become an indispensable part of intensive care medicine and is of great importance for patient care. In addition, ultrasound is included in the European and Dutch list of competencies to be acquired by intensivists in training. This document describes the frameworks within which ultrasound is embedded and performed in the adult intensive care unit of Amsterdam UMC, VU Medical Center.

## Definition

In this document, intensive care ultrasound (ICARUS) refers to combined heart and lung ultrasound used in relation to the diagnosis and treatment of critically ill patients.

In practical terms, this mainly concerns essential and relatively basic ultrasound scans of the heart and lungs, which are interpreted in conjunction with other available information about the patient.

In the literature, this is also referred to as bedside or point-of-care ultrasound (POCUS). Point-of-care ultrasound should therefore be clearly distinguished from and is not a substitute for specialized ultrasound as performed by cardiologists, radiologists, and gynecologists.

## ICARUS Committee

The ICV staff has established the ICARUS committee. It currently consists of: B.L. ten Tusscher, H.P.M.M. Gelissen, P.R. Tuinman, P. Thoral, P. Elbers. The ICARUS committee is the point of contact for ultrasound in the ICU.

## Certification

In order to acquire sufficient competence in cardiac/pulmonary ultrasound, all fellows must participate in the ICARUS program. Other physicians who have been working in the intensive care unit for at least six months, such as AIOS anesthesiology, may participate in the ICARUS program on a voluntary basis. If both course days have been completed, 40 full ICARUS ultrasounds have been documented, and the exam has been passed, the ICARUS certificate can be obtained.

## Status of ultrasound images produced

The term ICARUS ultrasound should only be used for distinction purposes if a complete ICARUS ultrasound has been performed. Ultrasound technicians who are not yet ICARUS certified must have the ultrasound assessed by an ICARUS-certified intensivist within 24 hours.

Only conclusions derived from the mandatory ICARUS recordings by certified

IC physicians may be used for clinical decision-making. NB: If a complete ICARUS ultrasound is not performed, but only limited recordings or recordings that fall outside the scope of the ICARUS ultrasound, these must be reported as point-of-care ultrasound and also assessed by an intensivist with expertise in the relevant field or a consultant in the relevant specialty, with the co-assessor also being named in the file.

## ICARUS program

The ICARUS program consists of a basic course and a consolidation course, including a practical component. Once both have been successfully completed, the participant can register for the ICARUS exam. Upon successful completion, a certificate will be awarded.

*1) Basic course day.*

This day includes an introduction with a brief discussion of basic ultrasound physics and the operation of ultrasound equipment. The remainder of the day is spent alternating between presentations on basic lung and heart ultrasound (the images to be taken and their interpretation) and hands-on sessions.

If another course has been taken, it can be discussed whether this basic course day can be waived and the candidate can possibly start immediately with the rest of the program.

### 1^e^Practical component (minimum of 10 ultrasounds)

After the basic day, the candidate can start performing ultrasounds in the intensive care unit. After performing the ultrasound, it is saved and uploaded to the patient file. Reporting takes place via the procedures/ICARUS tab. Non-certified sonographers must have the ultrasound performed assessed by a certified intensivist within 24 hours. Also make a short note in the daily status.

### 2) Consolidation day

The consolidation course consists of a ^second^course day with a brief theory review of the basic physics of ultrasound (expanded with Doppler principles) and cardiac and lung ultrasound. In addition, the presentations emphasize the integration of cardiac and lung ultrasound and interpretation in the context of other clinical information about the patient. Presentations are interspersed with hands-on sessions.

Starting in September 2019, the course will be gradually expanded. The first addition will be determining stroke volume and/or cardiac output via LVOT-VTI (Velocity Time Integral) and measuring Left Ventricular Outflow tract (LVOT) diameter. In addition, the principles of color Doppler imaging, Pulsed Wave and Continuous Wave Doppler will be introduced, along with their use in determining the presence of tricuspid (TI) and mitral valve insufficiency (MI).

If TI is present, the gradient across the mitral valve can be measured to obtain an indication of RV systolic pressure. Variability in the flow across the mitral or tricuspid valve can be used as an additional echo parameter in cases of suspected tamponade based on clinical findings and other ICARUS observations such as pericardial effusion, compression of the RA and/or RV, and an increase in the diameter of the IVC. For the time being, these additional recordings are optional.

### 2^e^ Practical part (remaining 30 echocardiograms)

These ultrasounds must be of a sufficient standard (comparable to that of a certified sonographer in terms of image quality and interpretation). This is at the discretion of the certified intensivists. The physician to be certified is responsible for demonstrating compliance with these qualitative and quantitative requirements.

## Handling the ultrasound machine

It goes without saying that the sonographer is responsible for cleaning the ultrasound device properly. This is of the utmost importance, out of courtesy to the next sonographer, but above all to prevent cross-infections. After use, first remove visible contamination with gauze, then clean with oxywipes from the clean side (cord) to the dirty side (of the probe). N.B.

Cords must not be cleaned with alcohol-based liquids.

Call each other out on not leaving the equipment correctly; in the event of a repeat offense, a participant may be excluded from the ICARUS program in extreme cases. The ultrasound device must always be returned to the

correct location and department to enable images to be uploaded. The plug must be inserted into the power outlet. The device may be left on to reduce the start-up time. However, the screen must be closed.

After uploading, remove the ultrasound device from the device.

If the ultrasound device is found to be defective or becomes defective during use, report this to: ICARUS@amsterdamumc.nl

## ICARUS Basic recordings

A distinction is made between mandatory and optional recordings. Below is an overview of these recordings, including practical instructions. Even if the mandatory recordings seem technically difficult or impossible, the best possible clip must be recorded. Background information about the recordings can be found in the literature references, among other places. A practical guide is also included at the end of this document. **ICARUS – Recordings and Practical Instructions**

- Lung and pleura:
- Blue points: linear vascular probe; if in doubt about B-lines, confirm with a cardiac phased array probe (with all filters disabled/lung setting). Select the correct depth: pleural line at approximately 1/3 of the image
- PLAPS: cardiac phased array probe.
- Heart and vessels: cardiac phased array probe
- Choose the right depth: the heart should occupy approximately 2/3 of the image
- Record clips of all views (acquire) except for the M-mode views
- For M-mode views: record a still image (🡪 acquire)
- For Doppler mode: record a still image with the measurements, for calculations, also save the measurement.
- Always record all windows, even if the images are poor
- Type a clear label for each window, unless it is obvious what is being displayed. Also consider left/right

## Lung and pleura

| **Mandatory** |  | **Optional** |
| --- | --- | --- |
| **Linear probe / lung setting** |  |  |
| 1. Upper Blue (1) left 2D mode | •  • | If lung sliding is absent: try to identify the lung point  Also scan the other intercostal spaces if there is sufficient time. |
| 2. Upper Blue (1) left M-Mode |  |  |
| 3. Lower Blue (2) left 2D mode |  |  |
| 4. Lower Blue (2) left M-Mode |  |  |
| 5. Upper Blue (1) right 2D mode |  |  |
| 6. Upper Blue (1) right M-mode |  |  |
| 7. Lower Blue (2) right 2D mode |  |  |
| 8. Lower Blue (2) right M-mode |  |  |
| **Cardiac probe / cardiac setting** |  |  |
| 9. PLAPS left |  |  |
| 10. PLAPS right |  |  |

Explanation of optional:

Lung point: only present in partial pneumothorax. Lung points are easier to find if the probe is rotated 90° from perpendicular to the ribs to the longitudinal direction of the intercostal space.

Scan of the other intercostal spaces: The 4 blue points enable rapid assessment of an acutely dyspnoeic patient, but minor abnormalities may be missed.

## Heart and vessels

| **Mandatory** | **Optional** |
| --- | --- |
| **Parasternal window** | |
| 1. PLAX | Measurement of LVOT diameter |
| 2. PSAX-AV |  |
| 3. PSAX-MV |  |
| 4. PSAX-mid Papillary muscle |  |
| **Apical window** | |
| 5. AP4C 2D mode | Color Doppler TV and MV |
| 6. | TI gradient (RVSP) (CW Doppler) |
| 7. | TV or MV flow variation (PW Doppler) |
| 8. AP4C M-mode (TAPSE) |  |
| 9. AP5C | LVOT-VTI (PW Doppler) |
| 10. AP2C |  |
| 11. AP3C | Alternative LVOT-VTI (PW Doppler) |
| **Subcostal window** | |
| 12. SC-4C |  |
| 13. | SC-SAX |
| 14. SC-VCI M-mode (VCI diameter and VCI collapse %) |  |

Explanation of optional:

LVOT diameter: for CO determination using LVOT-VTI and heart rate Color Doppler TV and MV: determine whether there is insufficiency of the tricuspid and/or mitral valves. Do not quantify, but determine the position of the maximum jet.

TI gradient: if TI is present in the maximum jet, use CW Doppler to determine the maximum flow and use this to calculate the pressure gradient. Supplemented with the CVD, this gives the RV systolic pressure (RVSP). TV or MV flow variation: If tamponade is suspected. Use PW Doppler to measure the flow across one of the valves. A variation in the height of the E wave greater than 30–35% supports the diagnosis.

## Indications

It is reasonable to obtain additional information via ICARUS once for each intensive care patient, preferably as early as possible during admission. For follow-up ICARUS examinations, the indication must be stricter, for example, unexplained cardiopulmonary deterioration or the evaluation of therapy. Therefore, for a follow-up ICARUS, the indication must first be discussed with the supervisor.

## Reporting

At VUmc, the images are stored and can be found in EPIC in the patient's results after uploading (via results/other/open latest CR thorax/select show images/click again on CR thorax, after which all imaging studies will be displayed, including ICARUS results). Reporting takes place in EPIC via Procedures/Procedure notes/Create note – select ICARUS. The report is based on the mandatory recordings that will be explained further below. The report must include at least the following points:

1. Patient's name, number, and date of birth
2. Reason for ICARUS examination (possibly: training)
3. Name of ultrasound technician
4. ICARUS certification status of the sonographer
5. Name of ICARUS supervisor and date of supervision (or: to follow)
6. Lung sliding present/absent on the left
7. Lung sliding right present/absent
8. PLAPS left present/absent
9. PLAPS right present/absent

10.Blue Profile A/B/C

11.Diagnosis according to BLUE

12.LA normal/dilated

13.RA normal/dilated

14.LV normal/dilated/hypertrophic

15.RV normal/dilated/hypertrophic

16.LV function poor/moderate/good/hyperdynamic

17.Measured LVOT diameter,

18.Measured LVOT VTI, measured heart rate, calculated CO and SV

19.Measured TAPSE

20.RV function poor/moderate/good/hyperdynamic

21.Color Doppler indication for TI present/absent

22.Color Doppler indication for MI present/absent

23.Measured gradient across the tricuspid valve

24.Measured or estimated CVD

25.Estimated RVSP

26.Pericardial effusion yes/no

27.Measured E-wave variation (%) across mitral or tricuspid valve

28.Suspected tamponade no/minor/major

29.Measured IVC diameter, measured IVC collapse (%)

30.Filling status hypovolemia/normovolemia/hypervolemia

Cardiac output (CO): for follow-ups of Cardiac Output (CO) in successive ICARUS echocardiograms, the first measured LVOT diameter must always be used! This is an anatomical measurement that does not change. Check the LVOT diameter, but only adjust it if you think that this first measurement may be incorrect. If you do adjust it, make sure to note this in your report.

## Choice of ultrasound probe

For lungs and pleura, good results can be achieved with the linear vascular probe (>10 MHz), the cardiac phased array probe (1-5 MHz), and the curvilinear abdominal probe. In general, high resolution comes at the expense of depth, and vice versa.

The cardiac phased array probe is best used for the heart and vena cava.

However, in emergencies, the curvilinear abdominal probe can also be used. Naturally, this probe is also preferred for abdominal structures, including the abdominal aorta.

# Lungs

When performing ultrasound of the lungs and pleura, it is very important that all filters on the ultrasound device are deactivated. On the SonoSite EDGE-II, this is done by selecting the lung setting. This is because ultrasound is used to detect artifacts, which should not be suppressed. For other applications, the corresponding presets can be used.

## Position of the probe for lungs and pleura

The patient is in a supine position. The so-called blue and PLAPS points are used.


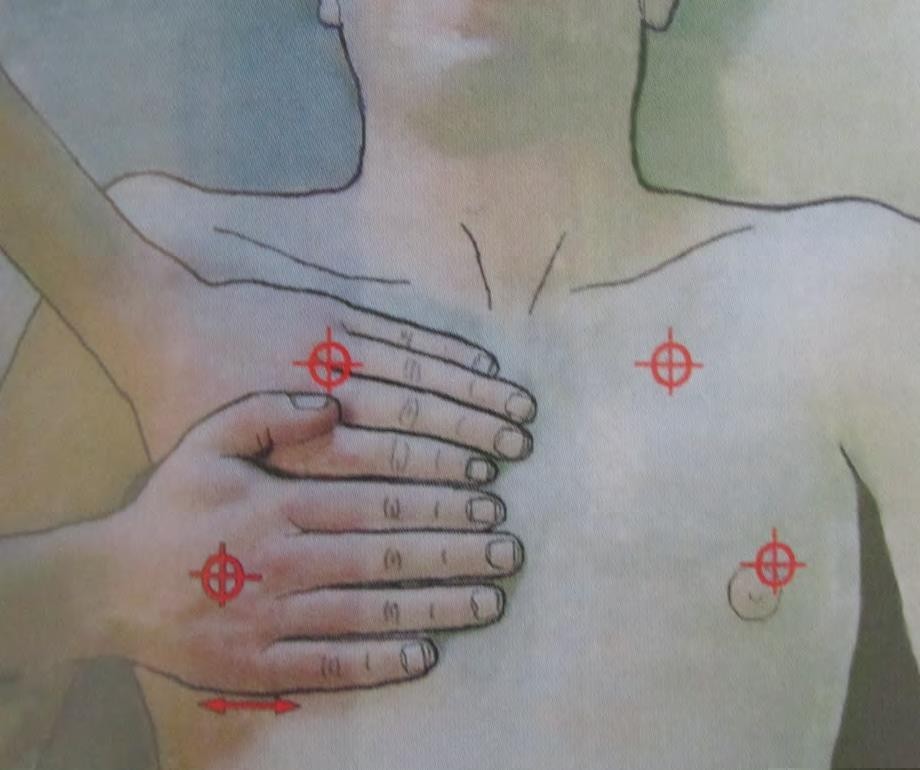


The blue points can be found by placing both hands on the thorax as shown in the figure. Blue 1 is located between the ring and middle fingers of the cranial hand at the transition to the palm. Blue 2 is located in the middle of the palm of the caudal hand.

Therefore, Blue 1 is the most cranial and Blue 2 is the most caudal of these two points. Blue 1 crosses approximately the midclavicular line. Blue 2 is lateral to the midclavicular line, but medial to the anterior axillary line.

The echo probe must be in the sagittal plane and therefore perpendicular to the ribs, towards the upper part of the thorax. The convention is that the marker on the probe is directed cranially and the marker is on the left side of the image. The left side of the image thus corresponds to cranial. N.B. when used in a cardiac setting, the probe must be rotated with the marker pointing caudally.

PLAPS stands for Posterior Lateral Alveolar and/or Pleural Syndrome. The PLAPS point is located in the lowest part of the thorax. Again, the probe is positioned sagittally with the marker pointing cranially. Guided by the image, the probe is placed as laterally and posteriorly as possible, just above the diaphragm. An important goal is to search for pleural fluid, which is why the echo probe is positioned as dorsally as possible, scanning toward the anterior thorax and thus the ceiling.


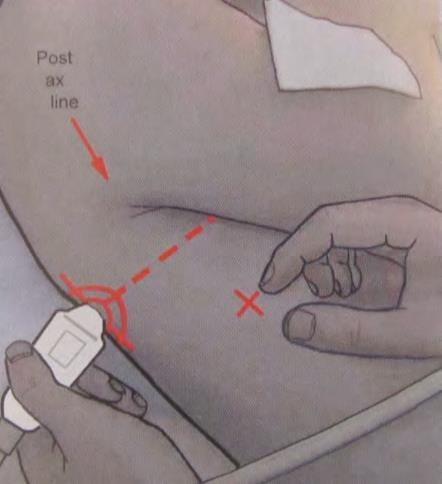


The lung point is optional and only exists in the case of a pneumothorax, and is therefore dependent on the findings in the mandatory images.

## Interpretation of ultrasound images of the lungs and pleura

During the assessment, one should look for the bat sign, A-lines, B-lines, lung sliding, alveolar consolidations, and pleural effusion. A- and B-lines are artifacts. In the ventral echo windows blue-1 and blue-2, look for artifacts (A and B lines), lung sliding, and alveolar consolidation. In the lateral dorsal echo window (PLAPS), look for posterolateral alveolar and/or pleural syndrome. That is, the combination of alveolar consolidation and pleural effusion. In the report, indicate whether positive PLAPS is accompanied by consolidation, pleural effusion, or both.

The bat sign guarantees uniform image production. The bat sign consists of the image of two ribs and their acoustic shadows and the pleura in between with the normal lung or pathological structures behind it. The bat sign is created by the bat figure formed by the white periosteal line above the ribs (wings of the bat) and the bright white line of the parietal pleura (body of the bat; in the adjacent lung, the white line is a combination of the parietal and visceral pleura).

A-lines: These are reverberation artifacts caused by repetitive reflection of the ultrasound between the probe and the pleura. Precisely at double, triple, etc. distances from the echo probe, one or more echoes of the pleural line can be seen. These lines therefore run horizontally (parallel to the echo probe). They are an indication of the presence of gas/air under the pleura. This can be either a pneumothorax or intra-alveolar gas/air, as seen in a normal lung.


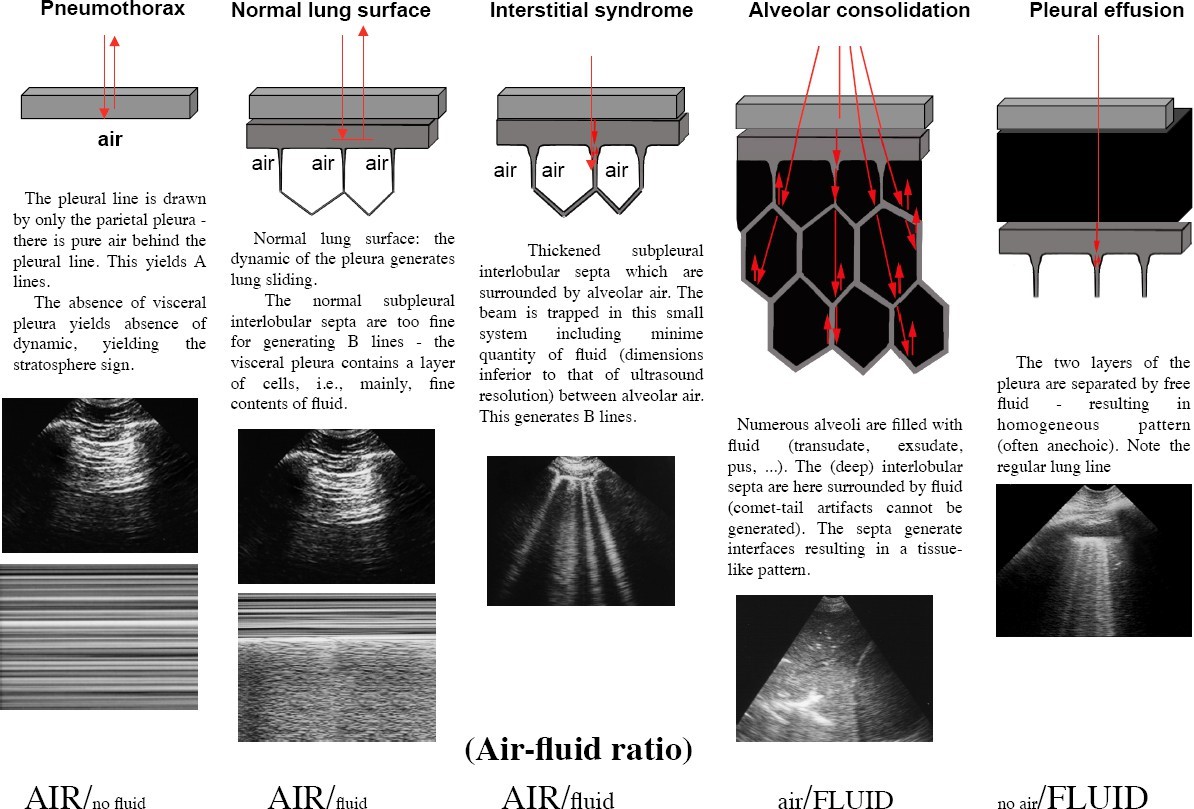


B-lines: These are artifacts caused by fluid. From the pleural line, very large tails can be seen extending deep into the lung tissue. Note: there are also other vertical artifacts besides B-lines. Important distinguishing features of Blines are that they blur A-lines and that they extend deep into the periphery. Therefore, when assessing B-lines, one must be careful with images from a vascular probe that provides less depth. B-lines are an indication of fluid in the alveoli

or the interstitium. More than three (3) such tails are referred to as B+ lines. The lines are always vertical and run away from the echo probe.

Lung sliding: this is the subtle sliding of the visceral pleura along the parietal pleura during spontaneous breathing or ventilation. It is often described as crackling. Sometimes small moving spheres can be seen in the pleura that have a tail pointing towards the lung tissue (comet tails). These may be Blines or other vertical artifacts. In M-mode, the presence of lung sliding can be seen as the seashore sign. The transition from lines (non-moving tissue) to noise (alveolar/lung tissue) is the location of the pleura. This is usually visible as a thick white line. If there is no lung sliding, there is no transition and the image resembles a classic barcode or stratosphere. However, caution should be exercised when interpreting M-mode images with regard to lung sliding. In severe dyspnea, the intercostal muscles can move and thus generate a false sea-shore sign. Therefore, M-mode should primarily serve as confirmation of the 2-D image.

Lung Point: Move the echo probe over the highest side of the thorax to find the boundary of a pneumothorax. This is the transition where the pleural layers separate. The location is searched for in 2D mode. Confirmation is obtained by recording the M-mode during breathing. Movement of the lung will also cause the separation site to move.


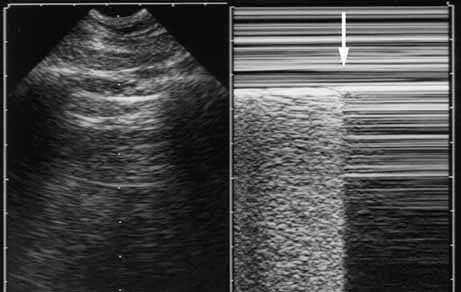


See the image above: The left image is 2D, the right image is M-mode. On the left side, the lung is attached (seashore sign); on the right side, the lung is detached (stratosphere sign). The arrow indicates the Lung Point.

Alveolar consolidations: In this case, the lung tissue in the 2D image appears as tissue filled with fluid rather than air, such as the liver or spleen.

Pleural effusion: Depending on the consistency of the fluid, this produces hypoechoic (usually) to hyperechoic images. Especially if the image is black, for example in the case of transudate, the fluid causes amplification of the image of the underlying structures (often consolidated lung tissue). In Mmode, displacement of the lung to and from the parietal pleura can be seen, with thickening and thinning of the pleural fluid layer; the sinusoid sign. Clotted blood, on the other hand, reflects the ultrasonic signal strongly (important for both the pleura and the pericardium!).

## Echo profiles and the BLUE protocol

The blue protocol was developed for the evaluation of blue patients. It can lead to a quick diagnosis and therefore treatment. That is why we report the ultrasound images of lungs and pleura within ICARUS according to this protocol. In order to gain a lot of experience in this, we also follow this method of assessment in patients who are not blue.

The profiles are distinguished based on the findings on the Blue and PLAPS points.

| A profile: | A-lines and lung sliding are both present. |
| --- | --- |
| A' profile: | A-lines present, no lung sliding and no lung point. B |
| profile: | B+ lines and lung sliding are both present. |
| B' profile: | B+ lines present but no lung sliding. |
| A/B profile: | B+ lines on one side of the thorax, A lines on the other side. |
| C profile: | Anterior/ventral alveolar consolidation |
| PLAPS: | Posterolateral alveolar and/or pleural syndrome, alveolar consolidation, and pleural effusion. |

Normal profile: A profile (A lines and lung sliding) present and PLAPS negative


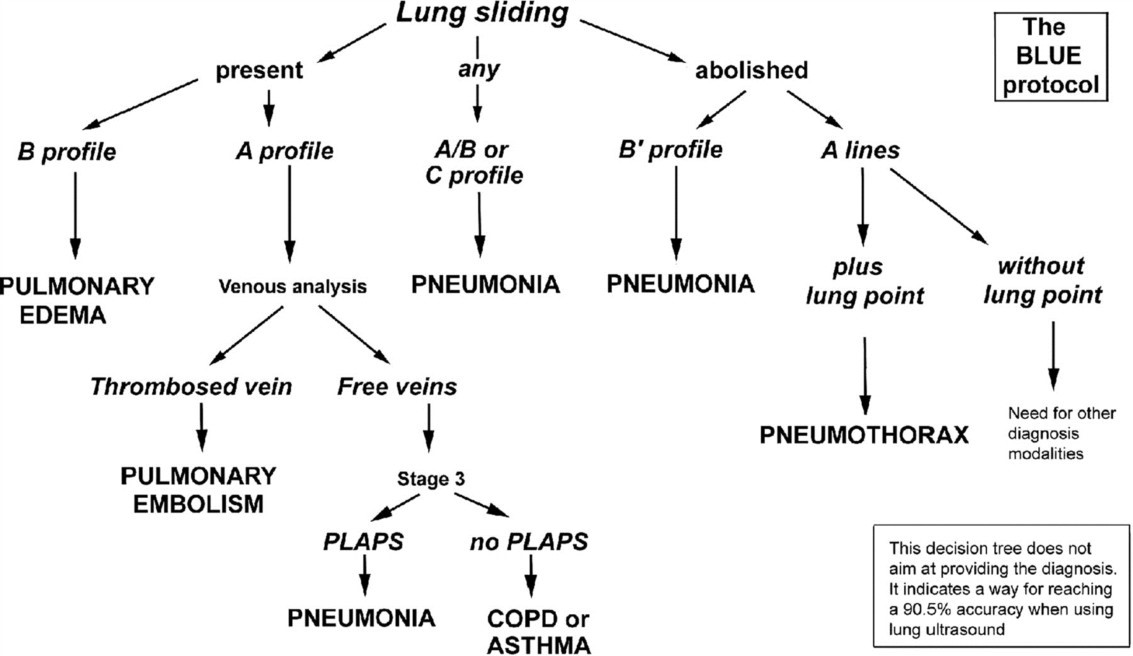


# Heart

## Position of the probe for heart and vessels

We follow the standard positions: parasternal, apical, and subcostal. Ideally, the patient lies on their left side (parasternal and apical windows) because this provides better images. Unfortunately, this is not always possible in an intensive care setting. In addition, positive pressure ventilation and PEEP sometimes result in the parasternal and apical windows producing images that are barely usable.

**Parasternal images.**

### **Parasternal long axis (PLAX)**

The parasternal images can be divided into parasternal long-axis (PLAX) and parasternal short-axis (PSAX) images. Below is an image showing the transducer position for the PLAX, which is always used to start with, and which structures can be seen. Classically, the parasternal window is located in the 3rd or 4th intercostal space, but this can vary.


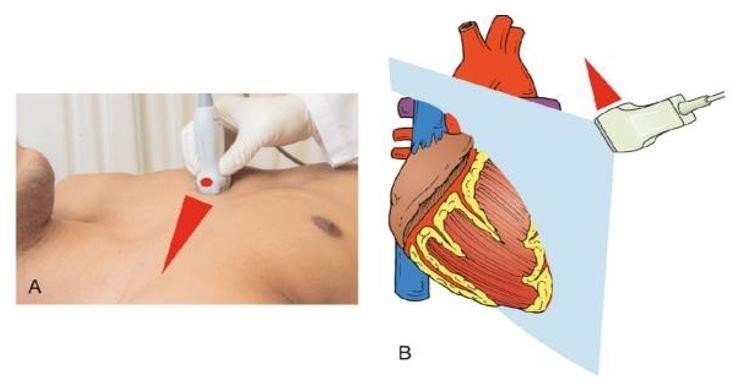

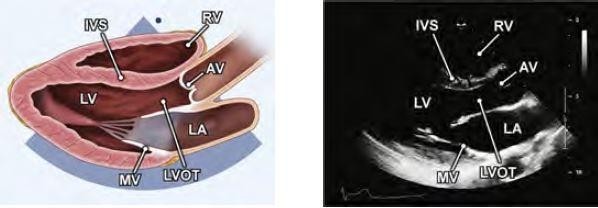


The following structures and functions can be assessed in this view. The right ventricle (RV, specifically its outflow tract RVOT in this view) and its function (RVF). Any pericardial fluid (especially in the right ventricle) and the degree of expansion of the RV. The anteroseptal part of the left ventricle (LV) and the posterior wall of the left ventricle (the image above is slightly rotated to show the inferolateral view).

An impression can also be obtained of the aortic valve (AV), mitral valve (MV), and left atrium (LA). The left atrium is normally no larger than 4 centimeters in diameter. The diameter of the left atrium is normally no larger than that of the aortic root.

If necessary, the end-diastolic thickness of the posterior wall can be measured. A thickness of up to 10 mm is normal, 10-12 mm is slightly abnormal or normal in young men who exercise frequently. A thickness of more than 12 mm indicates left ventricular hypertrophy (LVH). However, this measurement can only be reliably performed if a number of conditions are met: optimal recording with the ventricle fully open, good delineation of the myocardium, no papillary muscle in the image, M-mode perpendicular to the posterior wall.

***Optional:***

The LVOT diameter can be measured in the parasternal long axis. LVOT stands for Left Ventricular Outflow Tract. Assuming that the outflow tract is circular, the LVOT area can be calculated from the diameter using a simple formula (LVOT area (^cm²)^= Π x ^r²)^. Since the radius is squared in this formula, accurate measurement and optimal imaging (beware of foreshortening) are necessary. If necessary, zoom in, freeze, and then go to the midsystolic frame in which the aortic valve is fully open. Measure just before the insertion of the valve (maximum 0.5 cm from the aortic valve) from inner edge to inner edge.


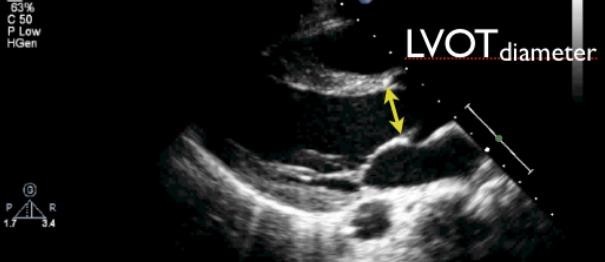


The stroke volume (SV) can be calculated from the LVOT diameter together with the LVOT VTI (see A5C) and multiplied by the heart rate to obtain the cardiac output. [CO = SV (= LVOT area x LVOT VTI) x HF].

If cardiac output is monitored by echocardiography, the first measured LVOT diameter is used for subsequent recordings, as this will not change. If the LVOT diameter cannot be measured reliably, it is possible to choose to monitor only the trend of the LVOT-VTI.

### **Parasternal short axis (PSAX)**

Rotating the transducer clockwise (90°) creates the parasternal short axis, which allows the heart to be viewed at different levels.

Below are images of the transducer position and the cross-sections.


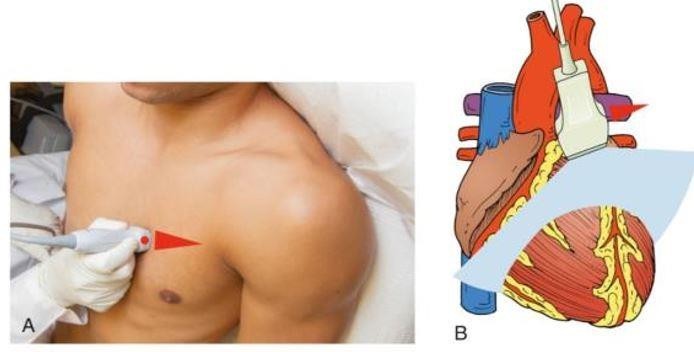


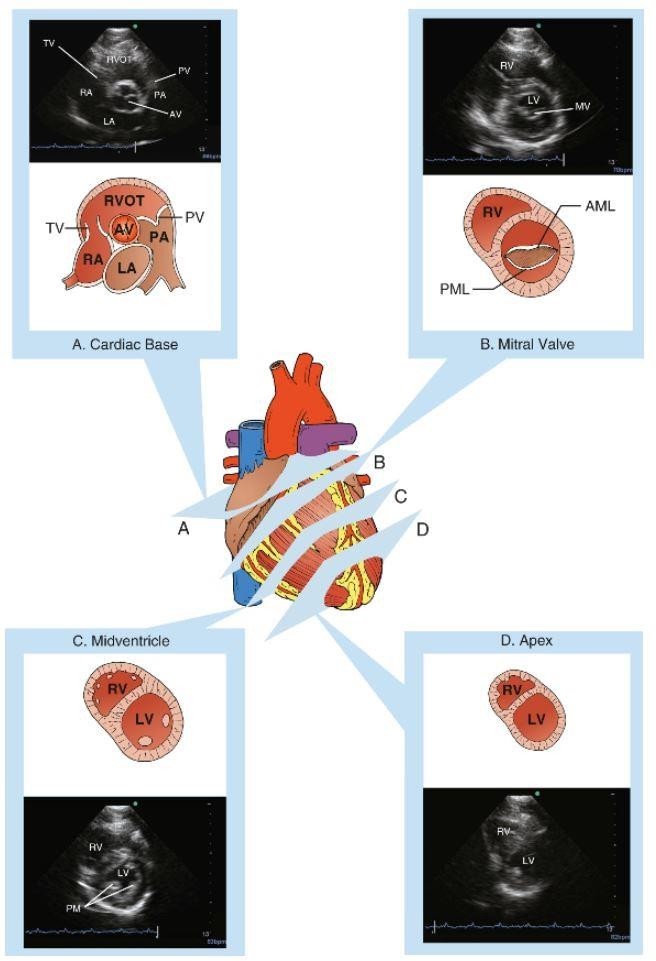


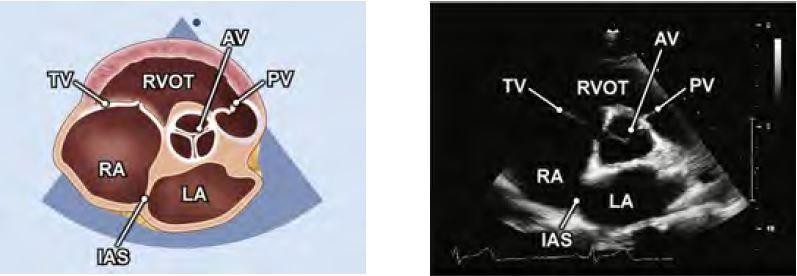


At section A, the aortic valve can be seen surrounded by the right system. In this image, the aortic valve is assessed for calcification and opening.


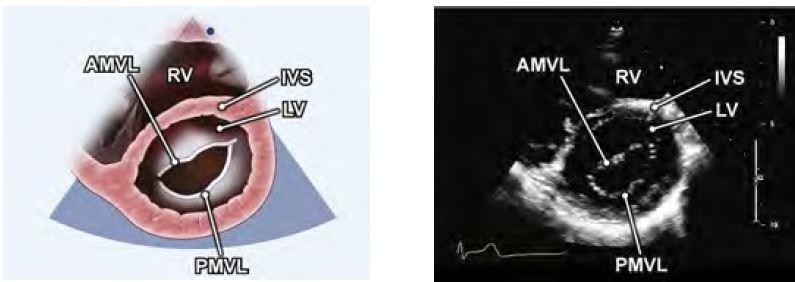


In section B, the mitral valve is assessed to determine whether it appears normal or abnormal, and its opening is assessed as normal or reduced.


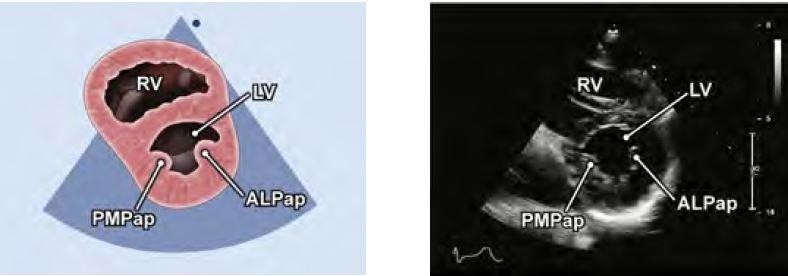


In section B and especially in section C, the left ventricular function is assessed. It is important for the intensivist to know that these sections allow you to assess (almost) all wall sections of the left ventricle.

The inward movement is examined in combination with myocardial thickening and the possible presence of regional differences in the various wall sections (cave asynchronous contraction pattern in conduction disorders, e.g., LBTB). It is important to note that the septum normally behaves as part of the left ventricle. Flattening of the septum may be consistent with volume (diastolic flattening) or pressure (systolic flattening) overload of the right ventricle. The size of the right ventricle can be better assessed in other images, because the angle at which the right ventricle is cut can have a major influence on the projection in the short axis images.

## Apical images

The apical images can be divided into the apical 4-chamber view (AP4C), the apical 5-chamber view (AP5C), the apical 2-chamber view (AP2C), and the apical 3-chamber view (AP3C).

# AP4C

Below you can see the transducer position for the AP4C and the structures that are visible.


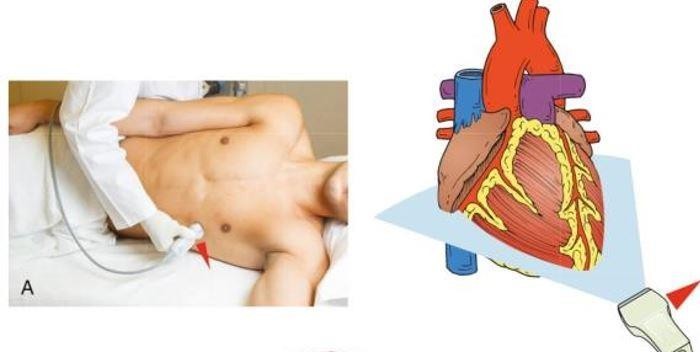


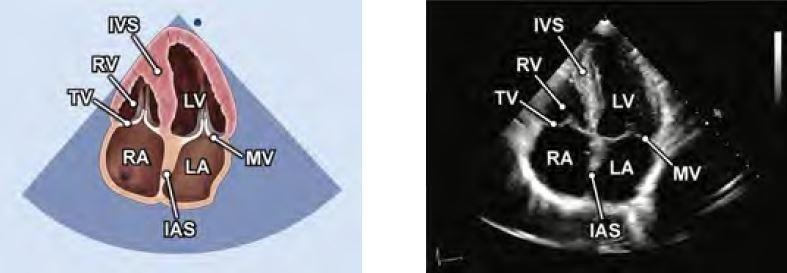


The AP4C is ideal for determining right ventricular function (RVF), both visually and using the so-called TAPSE. (Tricuspid Annular Plane Systolic Excursion). For the latter, the M-mode line is placed through the lateral suture of the tricuspid valve. This allows the distance over which it moves in systole to be measured. (Below 16 mm is abnormal, above 20 mm is normal, and between these values is indeterminate or moderate.)


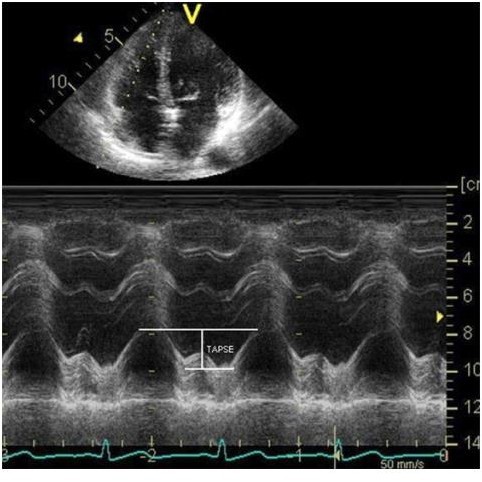


The septal and lateral parts of the LV can also be assessed. The dimensions of the right atrium (RA) and left atrium (LA) can also be assessed. In the case of

pericardial fluid, it is possible to see whether the right

system has collapsed. A collapsed right system then gives rise to a strong suspicion of tamponade (on echocardiography).

## *Optional*

CDI (color Doppler imaging) can be used in this view to visualize any insufficiency of the tricuspid and/or mitral valve, allowing the direction and size of any insufficiency jet to be assessed.

CDI makes use of the Doppler phenomenon. When a transmitted ultrasound pulse reaches a stationary object (or blood flow at right angles to the transducer), the echo will have the same frequency as the transmitted pulse. If the object/blood flow moves toward the transducer, the echo will have a higher frequency (positive Doppler shift), and if it moves away from the transducer, it will have a lower frequency (negative Doppler shift).

CDI uses Pulse Wave (PW) Doppler, in which short pulses of ultrasound are emitted. This allows blood flow to be recorded in a predetermined area, namely at a certain depth, between the horizontal lines on the Doppler line (sample volume).

With CDI, the direction and speed of the blood flow are displayed in color signals in the set color box, which are measured using PW Doppler along a large number of scan lines.

With PW Doppler, a short pulse of ultrasound is emitted and then received again (whereby the distance from the set sample volume to the echo probe determines the time in which the probe receives the reflection).

Before starting CDI, it is important to first optimally adjust the gain

(amplification) and depth of the 2D image. The color box must then be set as narrow and shallow as possible for optimal resolution and as parallel to the blood flow as possible to obtain an optimal Doppler signal.

Other important settings for CDI:

- first turn up the color gain and then turn it back down until speckle disappears.
- Color map: determines how flow is displayed in color.

The standard setting is that no blood flow is black, blood flow towards the transducer is displayed as red and away from the transducer as blue. Dark red means slow flow velocity and bright red to yellow means high flow velocity. In the case of turbulent flow, this is displayed by a mosaic pattern or green colors.

At higher blood flow rates and therefore Doppler shifts relative to the sampling frequency (Nyquist limit), aliasing occurs, causing the color to reverse. This is because the frequency of the transmitted pulses is too low relative to the blood flow rate. (As with a propeller that rotates faster and faster clockwise, after which it appears that the rotation is slowing down, stopping, and then turning counterclockwise, when in reality the propeller is rotating faster and faster clockwise). Aliasing can occur with any form of PW Doppler.


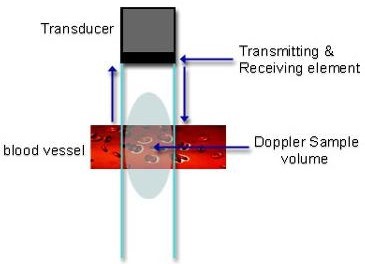

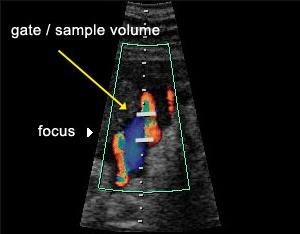


Spectral Doppler measurements use PW (pulse wave) or CW (continuous wave) technology. If the blood flow velocity is too high to be measured with PW Doppler, CW Doppler can be used. This involves continuously transmitting and receiving an echo signal (meaning there is no limit). However, the disadvantage is that all velocities along the entire scan line are measured. Since we use CW Doppler to measure the maximum velocity, this is generally not an issue.

In the presence of a tricuspid insufficiency (TI) jet, CW Doppler can be used to measure the velocity of the TI jet (TI velocity). This can then be used to quantify the pressure gradient across the valve using the following formula: 4 x (TI velocity)^2^. By adding the measured or, in the absence thereof, estimated CVD (see Table 1, page 32), we obtain the RVSP, which, in the absence of significant pulmonary stenosis, corresponds to the systolic pressure in the pulmonary artery.

# AP5C

The transducer is then directed slightly more towards the aortic valve to produce the AP5C. The corresponding cross-section is shown below.


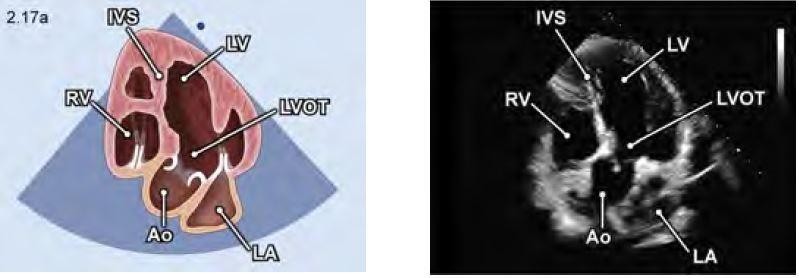


This image is called AP5C because it includes the aortic valve and the ascending aorta. Important to know: the aortic valve is closely related to the antero-septal part of the heart, making this image ideal for viewing this part of the heart. The other structures have already been seen in AP4C.

In the AP5C (and/or possibly the AP3C) recording, the VTI (velocity time integral) of the LVOT can be determined using PW Doppler. The direction of the Doppler cursor should correspond as closely as possible to the direction of blood flow in the LVOT and preferably be measured at the same distance from the aortic valve as the LVOT diameter (i.e., place the sample volume just before the aortic valve but not so close that flow acceleration occurs/there is no longer laminar flow with a set "gate" of 3 mm).


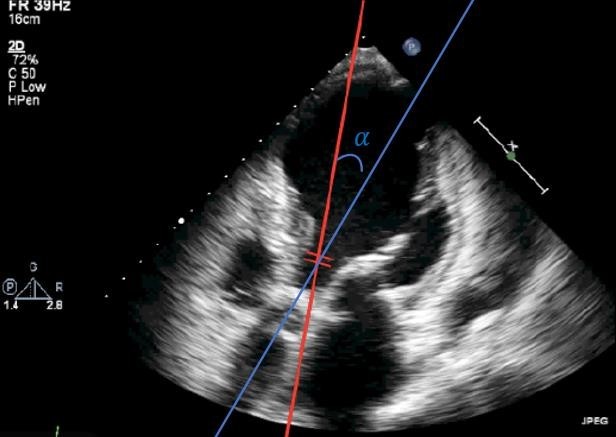


AP5C recording with placement of the sample volume of the PW Doppler in the LVOT.

If the angle of the Doppler signal deviates too much from the actual direction of blood flow, this will lead to an underestimation of the LVOT-VTI. If necessary, the angle can be adjusted on the screen (note that this does not change the quality of the signal, but the device recalculates the LVOT-VTI based on the specified angle).

If all goes well, this should produce an image as shown below. The flow direction is away from the probe, so below the baseline. NB: with laminar flow and a good signal, the envelope is not "filled in" as with CW Doppler, and with correct placement, the closure of the aortic valve is visible as a click. LVOTVTI is equal to the area under the curve and is calculated by the computer.

NB: sometimes it is possible to further improve the quality of the PW Doppler signal and the LVOT-VTI measurement by subtly moving the probe in the Doppler measurement setting in search of the 'best' signal.

Via Calcs/CO, go to LVOT-VTI and use select to "trace" the circumference of the VTI signal from the baseline (after aortic valve closure) back to the baseline. It is recommended to take the average LVOT-VTI from 2 to 3 beats at sinus rhythm and 5 to 10 beats at atrial fibrillation. The LVOT-VTI is unreliable in cases of pathological LVOT gradient (e.g., in very severe hypovolemia, SAM, etc.) or in cases of severe aortic valve insufficiency. If you determine the heart rate after the LVOT-VTI measurement, the computer calculates the CO (if an LVOT diameter has been stored previously). NB. With the

Sonosite machine, the heart rate must first be stored, i.e. before determining the LVOT-VTI.


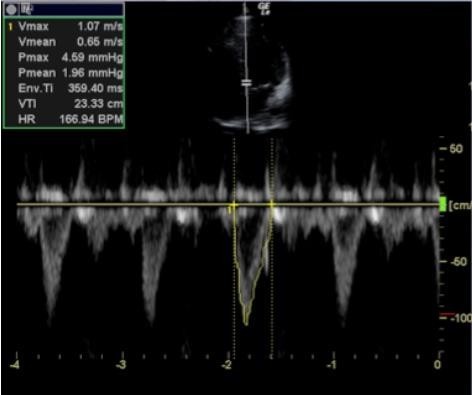


LVOT-VTI signal

LVOT-VTI is normally 18–22 cm at a heart rate of 55–95/minute [8,9]. Note that some articles use broader normal values of 15–25 cm. In any case, it is important to view the LVOT-VTI result in light of the heart rate, in other words, in the case of a heart rate slower than 55/minute, the LVOT-VTI must be greater than 18 cm, and if the VTI is 22 cm at a heart rate of 95, there is an increased stroke volume (SV) and cardiac output.

With successive LVOT-VTI measurements, the effect of interventions such as passive leg raising (PLR), a volume bolus, or the initiation of inotropics can be monitored. Since the LVOT diameter is constant, changes in stroke volume will be reflected by changes in LVOT-VTI. (Cave takes heart rate changes into account.)

Fluid responsiveness is often defined as an increase in stroke volume of >15% on a volume bolus and contractile reserve as an increase of 20%, whereby stroke volume can in principle be replaced by LVOT-VTI. In the event of hemodynamic instability, this can then be used as follows [8]:


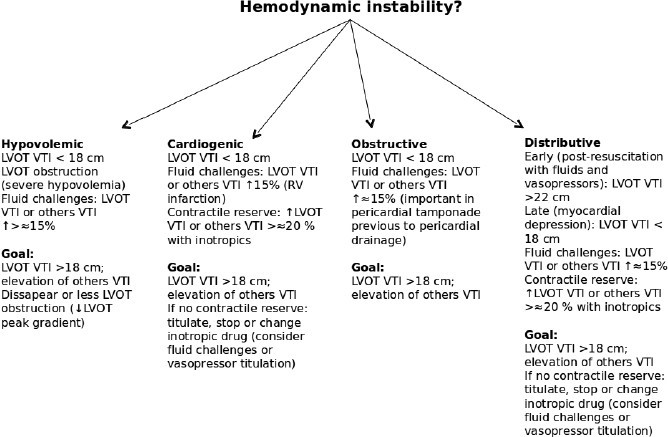


# AP2C

The transducer is then turned counterclockwise, creating the AP2C. The cross-sections are shown below.


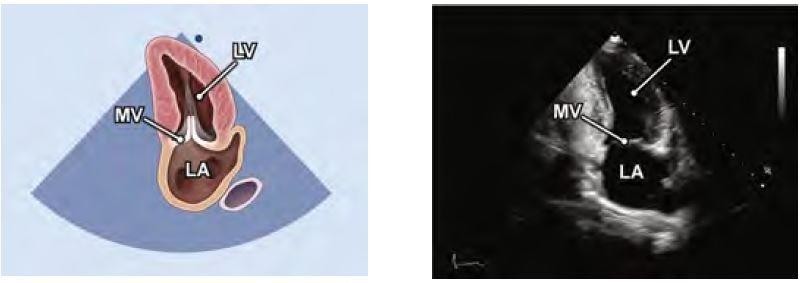


As can be seen, the inferior wall and anterior wall can be assessed well with this image.

# AP3C

The transducer is turned slightly further counterclockwise, creating the AP3C.

The cross-sections are shown below.


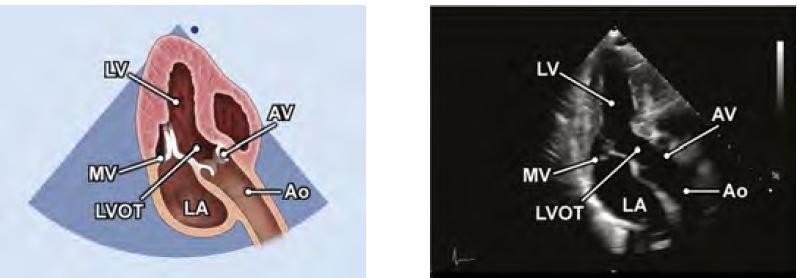


This image is particularly suitable for assessing the (antero)septal and posterior walls. The image corresponds to the PLAX, but is taken from a different position.

If a good LVOT-VTI signal is not obtained in the AP5C, it can be attempted in the AP3C.

## Subcostal images

Finally, a subcostal (SC) view is obtained. If patients do not have good parastinal and/or apical windows, it is advisable to quickly switch to subcostal because usable images can often be obtained there and because, with a little practice, virtually all ICARUS parameters that an intensivist needs to be able to assess can also be seen subcostally.

# SC-4C

Below you will find transducer position and images for the SC-4C.


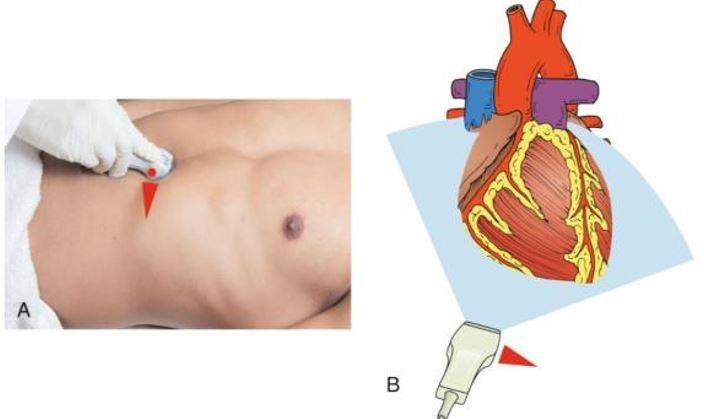

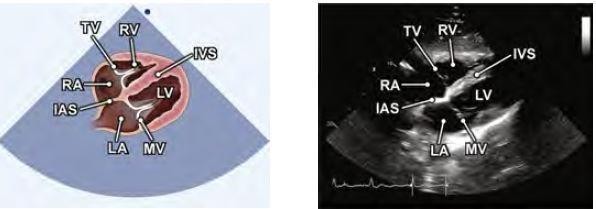


Pericardial fluid and the VCI can be clearly viewed in the subcostal window.

# SC-SAX

By turning the probe slightly counterclockwise from the SC-4C, you obtain the SC-SAX images. These are virtually identical to the PSAX images and are used as an alternative to them in cases of poor image quality.

# SC-VCI

To visualize the VCI, the transducer of the SC-4C image must be rotated 90° counterclockwise and viewed slightly deeper, which requires some practice. In non-ventilated patients, the VCI collapses more than 50% during inspiration; if this does not occur, overfilling may be present. For ventilated patients, the cut-off values are less obvious. You can check whether the VCI is 'FLAT' or 'FAT', whereby the former in a patient with a hemodynamic problem is an indication of underfilling and probable filling responsiveness, and the latter (FAT) argues against this.


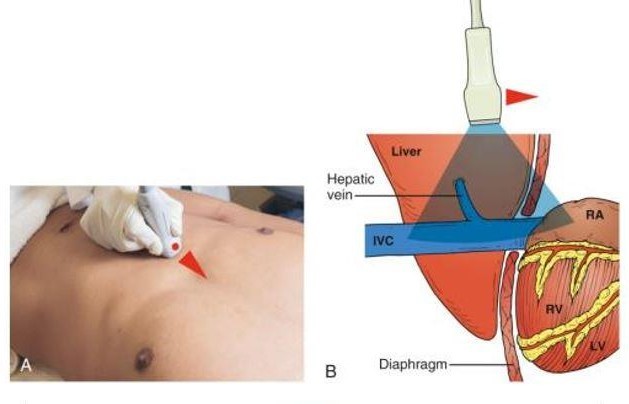

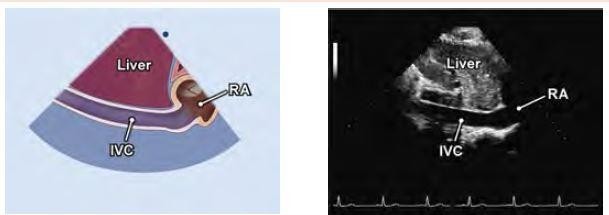


The diameter and degree of collapse of the VCI can also be used in nonventilated patients to estimate the CVD [10] if it cannot be measured, in which case the CVD can be used to calculate the RVSP. RVSP = 4 x (TI-vel)^2^+ CVD Table 1 [10]

| **RA pressure (mmHg)** | 3 (0-5) | 8 (5-10) | 8 (5-10) | 15 (10-20) |
| --- | --- | --- | --- | --- |
| VCI diameter (cm) | &lt;2.1 | &lt;2.1 | &gt;2.1 | &gt;2.1 |
| respiratory variation | &gt;50% collapse when sniffing | &lt;50% collapse when sniffing | &gt;50% collapse when sniffing | &lt;50% collapse when sniffing |

## Literature

1. Lichtenstein DA. Whole Body Ultrasound in the Critically Ill. 2010.

Springer, Berlin. ISBN 978-3540208228

1. Lichtenstein DA and Mezière GA. Relevance of Lung Ultrasound in the Diagnosis of Acute Respiratory Failure The BLUE Protocol. CHEST 2008;134:117–125

[http://journal.publications.chestnet.org/data/Journals/CHEST/22074/zcb00 708000117.pdf](http://journal.publications.chestnet.org/data/Journals/CHEST/22074/zcb00708000117.pdf)

1. International evidence-based recommendations for point-of-care lung ultrasound. Intensive Care Med. 2012;38:577-591 http://link.springer.com/article/10.1007%2Fs00134-012-2513-

4http://link.springer.com/content/pdf/10.1007%2Fs00134-012-2513-4

1. Levitov A, Mayo P, Slonim A. Critical Care Ultrasonography. 2009.

McGraw-Hill, New York. ISBN 978-0071592970

1. Soni, N. J., Arntfield, R., & Kory, P. (2019). Point of care ultrasound *(ebook)*. Elsevier Health Sciences.
2. Blanco, P., Aguiar, F. M., & Blaivas, M. (2015). Rapid ultrasound in shock

(RUSH) velocity-time integral: a proposal to expand the RUSH protocol. *Journal of Ultrasound in Medicine*, *34*(9), 1691-1700.

1. Sarti, A., & Lorini, F. L. (Eds.). (2012). *Echocardiography for Intensivists*. Springer Science & Business Media.
2. Rapid ultrasound in shock (RUSH). Blanco P et al. J of Ultrasound Med 2015; 34:1691-1700
3. Echocardiography for intensivists. Sarti A and Lorini F.L. (eds), DOI:

10.1007/978-88-470-2583-7. 2012

10)Rudski LG et al. Guidelines for the echocardiographic assessment of the right heart in adults: a report from the American Society of

Echocardiography 2010; 23:685-713
