# Supplementary material for: Thoracic Ultrasound–Related Management Change: Predictors and the Role of Operator Certification (Secondary Analysis of UltraMAN)
Source: J Clin Ultrasound. 2025 Oct 16;54(3):635–41. doi: 10.1002/jcu.70104 (PMC12967746; doi:10.1002/jcu.70104)

**Supplementary Figure S1.**

Forest plot of adjusted odds ratios (95% CI) for TUS-induced change in management from the multivariable logistic regression. An odds ratio >1 indicates higher odds of management change.

**Forest Plot: TUS-induced Change in Management**


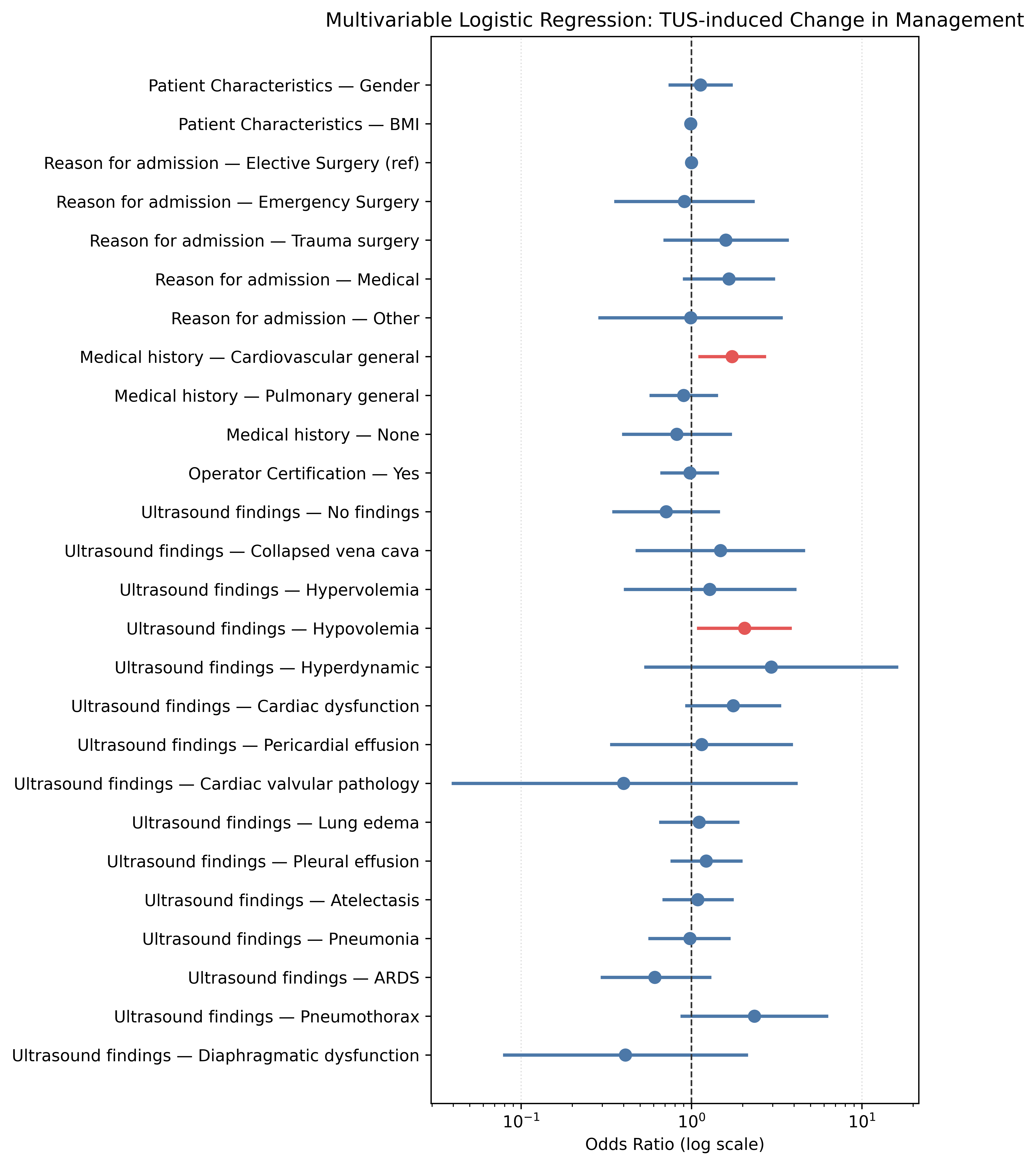

Supplement: Supplementary file 3 — Figure S1: Supporting Information. [file JCU-54-635-s001.docx]
